# Crossing Borders of Care: The Professionalization of Women in Nursing and China–US Collaboration at Xiangya, 1909–1926

**DOI:** 10.1111/nin.70119

**Published:** 2026-06-09

**Authors:** Yao Tang, Dominique Tobbell

**Affiliations:** ^1^ School of Nursing University of Virginia Charlottesville Virginia USA

**Keywords:** China–US medical cooperation, missionary nursing education, nursing professionalization, transnational collaboration, women in nursing

## Abstract

This article examines the development of modern nursing education in China through a case study of the Xiangya School of Nursing in Changsha between 1909 and 1926. Founded in 1911 by the Yale‐in‐China Association, a non‐denominational mission, Xiangya was among the earliest nursing schools in China to promote undergraduate nursing education and public health nursing. Drawing on archival materials, published primary sources, and Chinese and English historiography, we analyze the cultural, political, and gendered challenges that shaped the school's development under the leadership of American nurse Nina Gage. While Gage initially sought to implement an Americanized model of nursing education, she and her colleagues adapted admission policies, educational goals, and curricula to local social and cultural conditions. Recognizing the need for Chinese nursing leadership, they prioritized the establishment of a Bachelor of Science degree to prepare nurses for teaching, administration, and public health roles. By 1926, Xiangya had trained approximately 16% of China's qualified nurses and had become one of only two schools in China offering university‐level nursing education. This history highlights the interplay of transnational influence, cultural adaptation, and gendered reform in the professionalization of nursing in early 20th‐century China.

## 1 Introduction

In the early 20th century, the professionalization of nursing in Changsha, China unfolded through a complex interplay of transnational influence, cultural negotiation, and gendered reform. The Xiangya School of Nursing in Changsha, established by the Yale‐China Association (YCA) in 1911, serves as a pivotal case for examining how American and Chinese women and men collaborated in constructing modern nursing within a cross‐cultural environment.

Beginning in the mid‐19th century, with the expansion of Western empires and the consolidation of the treaty port system, medical missionaries arrived in China, establishing medical missions in several cities (Zhang 2023). In 1834, Peter Parker, dispatched by the American Congregational Church, arrived in Canton, China, and established a Christian ophthalmic hospital with a dedicated medical mission. This institution is recognized as one of the earliest Western‐style hospitals in China (Zhang 2023). Over the following decades, missionary organizations such as the London Missionary Society (UK) and the American Presbyterian Mission (USA) founded hospitals, clinics, and nursing schools in key coastal cities like Guangzhou and Fuzhou, as well as in international urban centers such as Shanghai (S. Grypma 2008). The Rockefeller Foundation also played a pivotal role in supporting medical and nursing education in China, notably through the establishment of the Peking Union Medical College (PUMC) in 1917 and its School of Nursing in 1920 (Allison 1993; K. Chen 1996b). These events contributed to the establishment of modern hospital‐based nursing in China and were part of the global expansion of modern nursing during the early 20th century.

The YCA was founded in 1901 by Yale University graduates with the goal of spreading modern education in China. Because of the limited missionary activity in central China, the YCA focused its educational efforts in the city of Changsha in Hunan Province. In 1905, physician and Yale graduate Edward Hume arrived in Changsha in 1905 to oversee the establishment of a hospital, which officially opened in 1908, and medical school, which opened in 1916. In 1909, Nina Gage joined him with the specific mission of founding a school of nursing. The hospital‐based training school admitted its first cohort of students in 1911. In 1926, Xiangya School of Nursing introduced a baccalaureate degree program in nursing, making it one of only two schools in China at that time to provide university‐level nursing education (Holden 1964, 140). Hume led the hospital and medical school and Gage led the nursing school until their departure from China in 1926 amid rising nationalism and the ensuing exodus of missionaries and other foreigners, laying the foundations for one of the most influential centers of modern nursing education in early 20th‐century China. Following their departure, leadership transitioned to Chinese faculty, and in 1929, Zigan Wang, a Chinese male physician, became the dean of the nursing school while also serving as the director of Xiangya Hospital (He and Huang 2014, 41).

As an early example of a university‐based School of Nursing in China, Xiangya is a valuable case study for analyzing the influence of transnational exchanges on the development of education and professionalization of nursing in modern China. Historians have primarily focused on the influence of such Chinese and US collaboration in coastal and internationally engaged cities such as Beijing (Jiang 2016), Shanghai (Jiang 2016), Guangzhou (Jiang 2016), and Shandong (Lu et al. 2018), leaving central regions like Changsha largely unexamined. An exception is Grypma's analysis of the role of Canadian missionaries in developing professional nursing in Henan province through the mid‐20th century (S. Grypma 2008). Despite being recognized by scholars as one of the notable nursing schools established around the same time as PUMC, the Xiangya School of Nursing has not received sufficient case‐level analysis in Chinese nursing historiography.

As a non‐denominational mission focused on educational rather than religious activities, the YCA contrasted with other prominent North American and British mission boards in China in the late 19th and early 20th centuries, which were denominational and pursued religious work (K. Chen 1996a, 1996b; Lei et al. 2014). In most cases, these mission boards had a separate Woman's Missionary Society that recruited, supported, and oversaw missionary nurses (S. Grypma 2008, 10). In contrast, the YCA was overseen by a Board of Trustees composed of Yale faculty, administrators, and alumni, which was ultimately responsible for the recruitment, support, and oversight of nurses at Xiangya (Holden 1964). While Christianity still infused YCA's educational work, it was not the primary factor in recruiting nurses and other staff. As Hume wrote in 1921, “What makes nursing worthwhile in China is the opportunity of bringing, in a Christian spirit, the ideals of the profession to a country hitherto devoid of even the term nurse. Our Trustees make no requirements of creed, but they desire to appoint to the staff only those who are actuated by a deep and sustaining Christian motive” (Hume 1921). For the American nurses recruited to Xiangya, their participation in religious activities was always voluntary and religious conversion was never part of their remit. In this way, Xiangya School of Nursing was distinct from the many nursing schools established by missionary boards in China in the early 20th century. Instead, it was structurally similar to the PUMC School of Nursing and, like PUMC, was an early advocate of both undergraduate nursing education and public health nursing (Allison 1993; K. Chen 1996b). But unlike PUMC, whose nursing program admitted only female students, Xiangya admitted male students during this period, reflecting a different vision of professional nursing education in China (Allison 1993). The history of Xiangya School of Nursing thus offers a nuanced perspective on the influence of transnational cooperation on the development of modern nursing in China in the early 20th century.

Drawing upon primary sources such as the YCA collections housed at Yale University and Edward H. Hume's memoir *Doctor East, Doctor West* (Hume 1946), as well as secondary scholarship on Chinese and American nursing history, we demonstrate the cultural, political, and gendered challenges that shaped this process. While Gage sought to implement an Americanized model of nursing education at Xiangya and elevate the status of nursing in central China, when confronted with realities on the ground, she and her colleagues adapted admission policies, educational objectives, and the curriculum to better meet the needs, norms, and priorities of the local community in Changsha. This included prioritizing the establishment of public health nursing to better meet the community's health needs and to increase local support for modern nursing. At the same time, Gage—with input from American nurse leader M. Adelaide Nutting—also experimented with curriculum reforms to mitigate some of the problems Nutting had identified in American nursing schools. Gage and her colleagues recognized the importance of preparing Chinese nurses for leadership and as such, prioritized the establishment of Bachelor of Science degree to prepare Chinese nurses for roles in teaching, administration, and public health, and in so doing, positioned nurses to play a key role in China's modernization project (Barnes 2018).

The article proceeds in three parts. First, it describes the context that led to the establishment of the Xiangya School of Nursing, including the work of the YCA and the role of the Nurses' Association of China (NAC), an organization that played a key role in the professionalization of nursing in China. Second, it examines the development and evolution of Xiangya's professional training system, including the school's admissions policies, educational objectives, curricular developments, and graduation standards, as well as the career trajectories of its early graduates, demonstrating how American ideas and ideals were adapted to local circumstances. Finally, it concludes by addressing the challenges, opportunities, and limitations of this transnational enterprise, revealing the influence of shifting political conditions, the advocacy of nursing students, and the complexities of cultural hierarchies and adaption on the transnational enterprise.

## 2 Establishing the Yale‐in‐China Association and Xiangya School of Nursing

Plans for the YCA were first formulated by a small group of Yale alumni in 1901, who were inspired by the broader missionary movement. Although initially conceived as a religiously based mission, YCA's founders ultimately decided it would be non‐denominational to better facilitate its educational mission (Holden 1964). During its evaluation of potential sites, the YCA concluded that major cities like Beijing and Guangzhou were already saturated with missionary activity. In contrast, central China, and particularly Hunan Province, offered a promising field for new missionary work free from existing competition. The YCA chose Changsha as the site for its educational institutions and hospital because the city combined strategic opportunity, social receptivity, and long‐term institutional potential. By the early 20th century, Hunan, though inland, was geographically well connected to Guangdong and Hubei and linked by waterways to the Yangtze River system. Changsha was an important commercial center, particularly a hub of the salt trade, with a population of over 2 million in the province. Its growing economic vitality suggested both material sustainability and a broad population in need of modern medical services. Equally important, new treaty arrangements had opened the interior to foreign residence and work, reducing earlier restrictions on missionary activity (Hume 1946, 7–8).

In 1906, YCA opened Yali Union Middle School to provide 4 years of secondary education to Chinese students with the goal of opening a college once there were enough Middle School graduates to attend. This they did in 1914, admitting the first students to the College of Arts and Sciences that fall (Holden 1964). In 1919, the Connecticut legislature passed an act that incorporated YCA under the laws of the state, giving the YCA degree‐granting authority (Holden 1964, 116–117). Reflecting its approach to religious activities, the YCA endorsed students having “full liberty to practice their own religion” (Holden 1964, 49).

The YCA regarded the provision of medical service—and with it, medical and nursing education—as part of its educational remit. Hume arrived in Changsha in 1905 to oversee the establishment of a hospital, which officially opened in 1908, and medical school, which opened in 1916. In 1909, Nina Gage arrived in Changsha with the goal of establishing a school of nursing. By this time, several American and British mission boards had established a handful of nursing schools in central China, but these early forays in nursing education were small, limited in scope, and short‐lived (K. Chen 1996a, 132–133). Gage taught the first cohort of students in 1911 and in 1913, the Xiangya School of Nursing officially opened with one division for men, whose students would care for male patients at the Red Cross Hospital, and one division for women, who would care for patients at the Xiangya Hospital (Holden 1964, 137–143).

The establishment of the Xiangya School of Nursing reflected the influence of transnational exchanges and feminist educational reforms. Gage, who had earned a bachelor's degree from Wellesley College and a diploma from Roosevelt Hospital School of Nursing in New York, was the younger sister of Brownwell Gage, dean of Yali Union Middle School (Levitan 2000). As a female American nurse working overseas in the early 20th century, Gage encountered a novel set of professional opportunities. At a time when the feminist movement in the United States was working to secure women's suffrage and intensifying its efforts toward the professionalization of nursing, missionary and other overseas nursing work offered American women unprecedented social legitimacy, career paths, and a degree of autonomy rarely offered at home (D'Antonio et al. 2013; S. Grypma 2008; J. Irwin 2013). As Gage reflected, “Yet one may do in mission work all these various kinds of work, the only difference being that abroad, one's opportunities are limitless, while at home one is more restricted” (Gage 1918). As historians argue, overseas work not only expanded access to professional autonomy for North American women but also strengthened nursing's legitimacy as a modern, international vocation (J. F. Irwin 2011).

Meanwhile, China was also undergoing transformative political and social change. The 1911 Revolution ended centuries of imperial rule and ushered in the Republic of China. Legal reforms following the revolution expanded women's access to education and suffrage (Yinhe 2011). Yet women's public education did not emerge abruptly in 1912. Late Qing debates had already begun to reconsider what women should learn and why, moving beyond household‐based moral instruction toward a more nationalized understanding of female education. By the early 20th century, the female student had become a visible symbol of modernity—short‐haired, unbound‐footed, and publicly mobile—representing both social progress and cultural anxiety. In this context, new avenues for women's professional training opened, nursing among them. Often framed within a form of “modernizing conservatism,” women's education was promoted not only as emancipation but as a means to strengthen family harmony, social order, and national prosperity (Judge 2008). Qiu Jin, born in Shaoxing, was among the educated women of this era. In 1907, she founded *The Chinese Women's Journal* (中国女报), which advocated women's participation in public life and gender equality. During her studies in Japan, she translated *A Textbook of Nursing* into Chinese, positioning nursing as a modern profession suitable for women and aligned with national regeneration (Andrews 2001).

Although the 1911 Revolution brought new opportunities, founding a nursing school in Changsha still faced many challenges. Traditional Chinese healing systems were deeply embedded in familial structures, with care often administered by female relatives, traditional practitioners such as the *san gu liu po* (三姑六婆, traditional female caregivers from local communities), or paid male attendants (Chung‐Tung 1991). Prevailing gender norms further stigmatized women's public roles and constrained their educational opportunities (Yinhe 2011). Cultural misunderstandings were frequent and could prove dramatic, as illustrated by one patient who, upon entering a Western‐style operating room filled with white‐clad staff, fled in terror, associating the scene with traditional funeral attire and impending death (Hume 1946, 82). As a result, the nursing program became a site of cultural adaptation, where Western nursing practices were adjusted to fit Chinese traditions. For example, within a few years of its opening, the Xiangya School of Nursing catalogs indicate that students wore dark blue uniforms rather than white (Hunan‐Yale School of Nursing 1926). Xiangya Hospital also adapted it practices to local cultural expectations, changing their bedding to red: “They painted the hospital beds black, and covered them with red bedding that was pleasing to the Chinese eye,” as red symbolizes joy and good fortune in Chinese culture (Levitan 2000, 95).

When nursing students were first recruited to Xiangya School of Nursing in 1911, there was no established Chinese translation for the profession of “nurse,” creating delicate and often humorous scenarios of cross‐cultural (mis)communication (Jiang 2016, 22). For instance, Hume created a Chinese poster which advertised the new profession as “Scholars to Watch and Guard” (Hume 1946, 168). Because Hume most likely had an imperfect command of Chinese, this was probably a highly awkward, literalist translation of 看 (*kan*, to watch) 護 (*hu*, to protect/to take care of/to guard) 士 (*shi*, either generically referring to a man or specifically referring to a scholar). While Hume's assembly of meanings of individual characters was incorrect, his choice of rendering *shi* as “scholar” was significant. Historians of Chinese nursing assert that it was an intentional choice to elevate the status of a caregiver—traditionally an undignified position—to that of a scholar, which stands at the top of the Confucian hierarchical pyramid. Liu attributes this neologism to the England‐trained nurse Miss Elsie Mawfang Lyon (née Chung, 钟茂芳), who, after consulting with a sinologist, coined the related term 護士 (*hùshì*) in 1914 at the NAC's national conference in Shanghai (Chung‐Tung 1991).

The establishment of Xiangya School of Nursing was influenced by the founding of the NAC in 1909. Initially, the NAC was “loosely organized and somewhat uncertain in its direction,” but after the 1911 Revolution, missionary nursing leaders met in 1912 and 1913 and resolved that the NAC should develop national standards for nursing education in China (K. Chen 1996a, 133). Membership was open to both Chinese and foreign nurses who had received formal training, as well as to hospitals and nursing schools that met the NAC's educational and ethical standards (X. Chen and An n.d.). Gage served as NAC president from 1912 to 1914 (K. Chen 1996a). The NAC designed a national curriculum, produced standardized teaching materials, introduced a unified examination system, and set credentialing requirements for both schools and students. At the time, nursing students were taught in hospital‐based training programs that combined clinical instruction and didactic lectures and required students to provide the majority of nursing care in the hospital. The NAC established regulatory criteria in 1919 that tied the legitimacy of a nursing school to quantifiable indicators of the associated hospital, namely that it should have at least 25 beds, serve over 200 patients annually, and offer obstetrics and pediatrics services. The NAC expanded its influence through communication and representation. It organized national conferences beginning in 1914 and in 1920, launched the *Quarterly Journal for Chinese Nurses*, providing a dedicated platform for professional discourse. In 1922, the NAC was admitted as a full member of the International Council of Nurses (ICN), signifying international recognition of its regulatory role (Levitan 2000, 96–103).

## 3 Professional Training at Xiangya School of Nursing

With the opening of the Xiangya School of Nursing, Gage sought to implement an Americanized model of nursing education in Changsha. However, when confronted with realities on the ground, Gage and her colleagues experimented with new professional training models in Changsha, ranging from the negotiation of admission standards to the design of the educational program. This was aided by the degree of institutional autonomy Gage secured for the Xiangya School of Nursing. In 1919, Gage's appointment as Dean of Nursing by the YCA Trustees granted her administrative authority and a seat on the Governing Board of the YCA Trustees, thereby placing nursing leadership on equal footing with the other academic faculties. Gage later reflected that she could be “the first Dean of a School of Nursing in the world,” underscoring the precedent‐setting nature of this institutional recognition (Levitan 2000, 94). By comparison, in 1921, Wu Zheying became the first principal of a nursing school affiliated with the Red Cross Hospital in Shanghai. And in 1924, the leader of the PUMC School of Nursing was designated as both “dean” and “superintendent of nurses” (Jiang 2016). The Xiangya School of Nursing's independent budget, rare at the time, signaled an evolving model of financial and administrative self‐determination within nursing education (Hume 1920).

### 3.1 Admission

A crucial first step in constructing the training system at Xiangya was determining who could be admitted. In the early 20th century, admission of nursing students was shaped by gender norms in China. At this time, men constituted a significant proportion of the student body, a pattern distinct from that in the West, where most nursing schools did not admit men until the 1970s (S. J. Grypma and Zhen 2014). Before the introduction of modern nursing in China, caregiving service were primarily performed by male attendants and san gu liu po (Chung‐Tung 1991). When missionaries established hospital‐based training programs, they initially recruited men from lower social classes as assistants. Several structural factors also contributed to men's initial predominance in nursing school enrollment: men generally had higher levels of education than women; boys who graduated from mission‐run primary schools could directly enter nurse training; and traditional norms limited women's access to education and restricted them from caring for male patients (S. J. Grypma and Zhen 2014; Jiang 2016). The Xiangya School of Nursing's early admissions policies were shaped by these prevailing gender norms. Indeed, as Hume recalled, when the Xiangya School of Nursing admitted its first cohort in 1911, Gage initially considered the male students to be more promising, lamenting the social burdens faced by the female students (Hume 1946, 168).

Between 1911 and 1925, Gage sought to maintain a relatively high standard for admission to the school, despite external pressures to expand enrollment rapidly. In 1911, the initial admission standard required applicants to have completed middle school (Holden 1964). This standard, however, limited women's access to the nursing school. Prior to the 1911 Revolution, women's access to education was extremely restricted and shaped by both economic circumstances and parental attitudes toward female education. Assuming a girl began her studies in 1911, she would need to complete 6 years of primary and 3 years of lower secondary (middle school) education—meaning that it would have been no earlier than 1920 when the first cohort of middle school‐educated girls might have become eligible for such programs. This contributed to the shortage of candidates who could meet the formal admission requirements.

In 1914, in an effort to expand enrollment, particularly among female students, Gage lowered the admission standard to primary school graduation. Gage intended this as a temporary measure and by 1917, the school catalog already showed a preference for those with at least 1 year of middle school, although difficulties recruiting female students continued. Gage's cautious approach is evident in a 1920 letter to Hume, where she confessed, “I did not dare make middle school a prerequisite” acknowledging the limited educational access for women at that time (Levitan 2000, 65). To help mitigate the issue, the nursing school introduced a preparatory year focused on math and Chinese (Gage n.d.). By 1925, Gage was able to raise admission standards again; that year, middle school graduation became the formal requirement. Reflecting on Gage's approach, a YCA physician later commented that while her standards might have been “perhaps too high under the circumstances,” especially given China's urgent need for more nurses, Gage's unwavering emphasis on quality set her apart from her colleagues (Levitan 2000, 64–66).

The challenges Gage faced maintaining high admission standards were not unique to Xiangya School of Nursing. Overall, the quality of nursing student intake in China was relatively low. According to a 1916 commentor, there was a lack of standardized criteria both for hiring nursing staff into hospitals and for admitting students into nurse training programs. In certain facilities, nursing staff possessed minimal literacy skills; although they were capable of measuring temperatures and pulses, they often failed to document these measurements. At a nursing program in Nanjing, admission officially required completion of 4 years of middle school. However, within the school's first 6 years, only 5 out of 32 enrolled students had received even 2 years of secondary education (Watt 2004, 70). PUMC also set its admission requirement at middle school graduation, which consequently resulted in a very small number of admitted students (K. Chen 1996b).

### 3.2 Education

#### 3.2.1 Curriculum Development

Nursing curricula reveal what is taught and how nursing knowledge is conceptualized and systematically organized. At Xiangya, the curriculum was 3 years in length. In 1916, the curriculum included subjects such as internal medicine, pharmacology, and surgical nursing reflected the growing influence of biomedical science in shaping modern nursing (He and Huang 2014, 254) (Table 1). Notably, the bilingual curriculum—featuring both English and Chinese—highlighted the transnational nature of the program and the dual demands of serving local populations while aligning with international standards. The organization of student coursework also reflected prevailing gender norms. Female students studied obstetrics and gynecology, while male students received instruction in urology and andrology—an arrangement that conformed to the boundaries of traditional gender roles in healthcare education (Jiang 2016).

**Table 1:** Nursing program curriculum table (He and Huang 2014, 50).

First year	Weekly hours	Second year	Weekly hours	Third year	Weekly hours
English	2	English	2	English	2
Chinese	2	Chinese	2	Chinese	2
Hygiene	2	Pharmacology	2	ENT & Ophthalmology	2
Bandaging Practicum	1	Nursing	1	Surgical Nursing	
Nursing	1	Internal Nursing	1	Dietetics & Sanitation	1
				Obstetrics/Maternity Nursing (Female)	1
				Pharmaceutics (Male)	1
				Nursing Ethics	1
				Pediatric Nursing	1

The curriculum also reflected the increased demands and scientific requirements of laboratory‐based medicine. In 1921, for example, as the number of special diets increased, nurses were required to do more work in Xiangya Hospital's diet kitchen, and as physicians regularly conducted “special tests and experiments” on the wards, they required “intelligent cooperation on the part of the nurses” (Gage n.d.). That year, physics was introduced into the nursing curriculum, marking a significant step in strengthening scientific training, and the following year, students benefited from access to laboratory facilities in the newly constructed science building (Gage n.d.).

In 1922, M. Adelaide Nutting—then a member of YCA's Nursing Advisory Board—was formally appointed to guide curriculum development at the Xiangya School of Nursing (Nutting 1922). Nutting had just completed a report on American nursing education, which documented widespread curricular disorganization, excessive reliance on student labor, and significant losses of instructional time (Nutting 1912). In the correspondence, she questioned whether Chinese nursing programs should fully replicate the customs of large, highly organized Western institutions. Nutting subsequently proposed a revised nursing curriculum to rectify the significant time wastage (estimated at one‐quarter to one‐fifth) common in existing 3‐year hospital‐based programs. In Nutting's view, the improved course structure, potentially shorter (two‐and‐a‐half‐year) in overall duration, offered a larger scope of content, better organization, and more thorough teaching (Nutting 1922). This ensured students' time was dedicated to meaningful education rather than ancillary hospital tasks, resulting in a more effective learning experience. Nutting's vision extended further: she advocated a university‐level nursing program lasting 4–5 years. This advanced pathway would provide the academic rigor and scientific grounding needed for roles in teaching, public health, and leadership (Nutting 1922). This vision of professional expansion was further reinforced by Gage's successful establishment of undergraduate‐level nursing education at Xiangya School of Nursing.

The establishment of a bachelor's‐level nursing program at Xiangya unfolded between 1921 and 1926. In 1921, Gage wrote to a YCA Trustee in New Haven, “I have accepted our first nurse who is also working for a college degree!” (Levitan 2000, 65). Subsequent correspondence suggests ongoing institutional planning. In 1922, Gage noted that the “University School of Nursing” might be offered as a regular department (Gage 1922a). By 1923, she further clarified that students enrolled in the university track would receive scientific training in the college (Gage 1915). This early experimentation evolved into a more structured educational model by the mid‐1920s. The Nursing School catalog of 1924–25 explicitly introduced a combined 6‐year university course, through which students could obtain either a Bachelor of Science or Bachelor of Arts degree alongside a nursing diploma (Hunan‐Yale School of Nursing 1926). In 1925, Gage reported that two students had already begun taking courses as part of their college work, with plans to proceed to clinical training the following year (Gage 1925b). These developments indicate that the program had moved beyond preliminary planning toward partial implementation (Gage 1925a; Xiangya Board 1925). By 1926, the program had become more fully institutionalized, as evidenced by the catalog outlining a structured undergraduate curriculum (Holden 1964, 140; Hunan‐Yale School of Nursing 1926) (Table 2).

**Table 2:** Nursing curriculum for the diploma and bachelor's programs, 1926.

Program	Year	Class	Periods a week	Total hours
Diploma	Preliminary year	Anatomy & Physiology	2	60
		Chemistry	3	90
		English (record keeping)	2	60
		Principles & Practice of Nursing	4	120
		Biology & Bacteriology	4	60
		History of Nursing	2	30
		Personal Hygiene	2	30
		Ethics of Nursing	2	30
		Physics	3	45
		Pathology	3	45
		Elementary Materia Medica	1	15
	Junior year	Massage & Rubbing	2	10
		Materia Medica	3	45
		Diet in Disease	3	45
		Review of Anatomy	2	30
		Surgical Nursing	3	45
		Obstetric & Gynecological Nursing	3	45
		Medical Nursing	2	30
	Senior year	Medical Nursing	2	30
		Pediatric Nursing	2	30
		Ophthalmic Nursing	1	15
		ENT Nursing	1	15
		Mental Hygiene	2	30
		Special Discussions	1	15
		Public Health Nursing/Ward Supervision	1	15
Bachelor's degree	Year 4	Principles & Practice of Nursing (Lecture 2‐Laboratory 4)	4	
		Materia Medica (Lecture 1‐Laboratory 1/2)	1	
		Diet in Disease	2	
		Personal Hygiene	1.5	
		Surgical Nursing	1.5	
	Year 5	Medical Nursing	3	
		Obstetric & Gynecological Nursing	1.5	
		Pediatric Nursing	1.5	
		Eye/Ear/Nose/Throat Nursing	1.5	
		Mental Hygiene	1.5	
		Public Health Nursing	1	

In a comparative context, Xiangya's bachelor's degree program—created in between 1921 and 1926—was at the cutting‐edge of nursing education both domestically and abroad. In 1920, PUMC opened a nursing school to train future educators and administrators. Students completed 1 year at Yanjing University and 3 years at PUMC, earning a diploma and nursing license. In 1922, a 5‐year bachelor's program was launched jointly with Yanjing University (Watt 2004, 70). Although PUMC was the first to establish a bachelor's‐level nursing program in China, the Xiangya program, as Holden argues, “was the first under university control,” noting that YCA had an undergraduate College authorized by the Connecticut legislature to confer degrees (Holden 1964). This was an option unavailable at PUMC, as PUMC did not itself confer undergraduate degrees (Allison 1993). In the United States, one of the first undergraduate programs in nursing was created at Yale in 1923, the same year the School of Nursing was founded (Yale School of Nursing 2016). In Japan, a 4‐year undergraduate nursing college program was not established until 1951 (Sakashita 2018).

#### 3.2.2 Education Objectives

The educational objectives of the Xiangya School of Nursing reveal how the school envisioned the professional roles and future trajectories of its nursing students. In 1925, nursing and physician leaders at Xiangya articulated the dual professional aims of nursing education: nurses were to be trained not only for bedside clinical care but also for more advanced roles in public health, hospital administration, and nursing education (Brundage et al. 1924).

Gage placed particular emphasis on preparing nurses for public health work. She was influenced by Lillian Wald's pioneering work at the Henry Street Settlement in New York, where nurses delivered home visits, school health services, and community hygiene education to advance community health and urban reform (Fee and Bu 2010). In 1922, Gage wrote to the YCA's executive secretary, Herbert Vreeland, that public health was the “coming work for China” because “it can be done in the homes.” Gage noted that Chinese people were reluctant to embrace private duty nursing, because “they do not understand it. But they like a nurse coming in the home to tell them what to do” (Gage 1922b). As Vreeland's response indicates, the YCA's Trustees similarly recognized “the importance of [public health] in bringing our entire work in the college into closer contact with the Chinese.” In this way, public health nursing was a way to “reach into the community to meet real needs and to improve conditions,” and to receive “fuller cooperation and more adequate support from Chinese sources” (Vreeland 1922).

In 1922, the YCA recruited Maude Barton to lead Xiangya's Public Health Department and teach public health courses (Gage 1922b). Barton had a BA from Smith College and a diploma from the Massachusetts General Hospital School of Nursing. She had also completed public health nursing courses at Simmons College, and before joining Xiangya, had spent a year at PUMC (“Miss Maude Barton” 1922). In the school's 1923–1924 annual report, Gage attributed the hospital's growing number of patients to the efforts of the Public Health Department, which actively promoted hygiene and disease prevention. In addition to Barton, the department was staffed by three nurses, including—by May 1923—two Chinese nurses (Gage 1923), who conducted follow‐up visits, social service inquiries, and health inspections in orphanages, schools, and poorhouses. Gage praised it as “one of the most constructive parts of our work,” particularly for reaching underserved communities. Its collaboration with the Hunan Poor Relief Association, whose adoption of its sanitary recommendations underscored effective inter‐agency cooperation, further reflected its impact (Gage 1924). In her 1926 article “The Nurse Enters College” (Educational Review), Gage stressed that in addition to bedside care and public health, nursing education should also include school and industrial nursing, and mental hygiene (Gage 1926).

PUMC also became an early laboratory for public health nursing in Republican China. In 1925, working with the Beijing municipal government, PUMC established a public health station where nurses conducted family visits, school and factory inspections, and hygiene education (Watt 2004, 73). This work reflected what historian Ruth Rogaski describes as China's emerging “hygienic modernity,” a project that linked national strength and social progress to improved sanitation, disease prevention, and everyday hygienic practice (Rogaski 2004). School health, in particular, was emphasized for its ability to improve children's personal hygiene and reach families through them; by 1930 the station's nursing staff had expanded from 6 to 17 nurses (Watt 2004, 73).

As Nicole Barnes argues, by the onset of China's War of Resistance against Japan in 1937, the gendered responsibilities of women to care for the hygiene and health of families and societies led public health nurses to occupy expanding roles within the healthcare system and the modernizing state (Barnes 2018). These developments in China also paralleled broader global trends in public health during the early 20th century as similar movements unfolded in places such as Poland and the Philippines, where American nurses helped circulate public health ideals and adapted them to local conditions (J. F. Irwin 2011).

#### 3.2.3 Faculty Recruitment and Language Policy

The recruitment of faculty and the choice of instructional language became key sites where transnational educational models were translated into local practice. As the Xiangya School of Nursing expanded, the demand for qualified nursing educators grew rapidly. Yet in the early 1920s, China lacked trained Chinese nurse educators. In response, YCA established the Nursing Board of Reference in 1920 to recruit qualified American faculty for Xiangya (“Memorandum Regarding a Nursing Advisory Board for Yale in China” 1920). Under the leadership of Nutting, the Board set up a nationwide recruitment network with representatives in major cities including New York, Philadelphia, Chicago, Seattle, and San Francisco. These representatives were responsible for identifying and interviewing nursing faculty to support the development of nursing education in China (Nutting 1920).

Language policies were emphasized in faculty recruitment. American nurses were expected to learn Chinese, completing a course of language instruction before undertaking teaching duties at Xiangya. For example, after arriving in Changsha in 1909, Gage spent an entire year studying the language (Hume 1946, 167). Requirements for Chinese‐language study were not uncommon at the time. For example, some missionaries in Henan were expected to undertake Chinese‐language study before assuming their duties (S. Grypma 2008). The emphasis on linguistic preparation was highlighted in the 1925 Nursing Report, which stipulated that foreign instructors could only teach with the assistance of interpreters if they demonstrated sufficient proficiency in Chinese. The report also expressed a clear preference for hiring qualified Chinese instructors whenever possible (Brundage et al. 1925). While instruction was conducted primarily in Chinese, students were required to maintain sufficient English literacy to read medical records and physicians' orders (Brundage et al. 1924).

Xiangya's language policy contrasted with PUMC's approach. At PUMC, nursing courses were taught entirely in English and followed the Johns Hopkins model. The program was rigorous, and only three students were admitted to the first class, two of whom later dropped out, partly because nursing was socially stigmatized as servant‐type work, and partly because the English medium of instruction posed a serious challenge (Watt 2004, 70). Xiangya's bilingual structure reflected both the practical realities of educating a largely local student body and the institution's broader goal of producing nurses who could function effectively within Chinese clinical settings. The contrast between the two schools' language regimes is significant. At PUMC, English proficiency served as a gatekeeping mechanism that limited enrollment to students with prior elite schooling. At Xiangya, the blended format reduced linguistic barriers and made nursing education more accessible. This difference illustrates how transnational nursing programs could take distinct forms depending on local needs and educational strategies.

#### 3.2.4 Local Leadership Cultivation

Establishing a sustainable training system required the cultivation of Chinese nursing leaders to assume leadership of the profession. By the mid‐1910s, Xiangya's leadership increasingly recognized that Chinese nurses—despite steady employment in hospital settings—remained underrepresented in educational and supervisory roles, limiting the school's long‐term prospects for Chinese leadership (Hume 1924). Gage sought to appoint competent Chinese graduates from Xiangya and other emerging nursing schools as instructors. However, she was unable to appoint Chinese instructors or supervisors during the early phase of the program as many potential candidates lacked adequate educational foundations and had limited experience in administrative roles (Levitan 2000, 92).

A major turning point in the process of indigenization occurred in 1917 when Cora Wong, a Chinese nurse trained in Manila and the United States was appointed to the faculty (Levitan 2000, 81–82). The following year, Edith Huang, a graduate of the Protestant Episcopal Hospital in Philadelphia was also appointed. Wong and Huang became the first American‐trained Chinese nurses to join the School of Nursing faculty. Their teaching in Chinese represented a crucial step toward transitioning from foreign‐led instruction to a more culturally embedded, locally sustained model of nursing education (Levitan 2000, 81–82). By 1925 YCA nursing policy explicitly acknowledged the ongoing short‐term dependence on foreign‐trained personnel, while also emphasizing the strategic importance of recruiting Chinese nurses educated abroad as a bridge toward the eventual Chinese take‐over of the school and hospital (Brundage et al. 1925). To institutionalize the goal of Chinese nursing leadership, in 1924, physician and nurse leaders at Xiangya recommended the appointment of a Chinese assistant superintendent of nurses (Brundage et al. 1925). This position was designed to foster local leadership and gradually transition managerial and instructional authority to Chinese professionals.

### 3.3 Graduation and Post‐Graduate Careers

As Xiangya's educational program matured, the standards for graduation and the career trajectories available to its graduates also evolved. In 1915, the NAC administered its first national examination for graduate nurses. Of the seven candidates, three passed, two of whom were from the Xiangya School of Nursing. Thereafter, Xiangya diplomas were issued only to those who successfully passed the NAC examination, reinforcing the NAC's role as a credentialing authority and aligning the school with emerging national standards (Levitan 2000, 98). By 1927, Xiangya had granted a total of 31 diplomas to men, and 36 diplomas to women (Holden 1964, 140). At the time, there were a total of 469 nurses across China that had passed the national credentialization exam initiated by NAC (Yan et al. 2014). Thus, Xiangya had produced about 16% of all nurses in China.

The career paths of the women graduates were notably diverse. According to the 1923–1924 annual report, six had married (presumably dropping out of professional careers to conform with gendered norms at the time), six were working in mission hospitals, two in public health, and two in Red Cross hospitals. In addition, one worked at the Isolation Hospital in Shanghai, five were employed by PUMC, one practiced private duty nursing in Peking, and two graduates were preparing to study in the United States on scholarships (Gage 1924). Men's disproportionate presence in early training cohorts was mirrored in their employment patterns after graduation. By 1924 Xiangya had graduated a total of 26 men nurses. Of these men, 15 went to mission hospitals, 3 to military hospitals, 3 to Red Cross hospitals, 1 entered medical school, and 4 fell ill (Gage 1924).

The employment of trained nurses had an immediate impact locally. The 1914 annual report observed that “the provision of trained nurses has at once resulted in a call for their services” in Changsha (Hume 1914). Mr. Shen Yu Chang, the first student to complete a 3‐year course under Gage, was steadily employed in private cases following his certification that September. Miss Wu, upon her graduation, was soon providing daily treatments to Lady Tang, the wife of the provincial governor and a patient of Dr. Yen, a doctor from Xiangya Hospital. Wu's specialization in obstetrics made her especially valuable in situations where a male physician was not desired (Hume 1914). The school, however, faced challenges in retaining graduate nurses to staff Xiangya Hospital. The 1921 annual report noted that four graduates had resigned that year to take positions at other hospitals. One left due to insufficient salary to support his family. Another sought further education, and her continued employment was uncertain unless the school could offer additional coursework in English, hospital management, and educational administration (Gage n.d.).

Graduates' employment opportunities were influenced by prevailing gender norms. Female graduates, especially those assigned to the homes of local officials, were frequently mistaken for domestic servants and faced dismissive or even hostile treatment. In some cases, they endured inappropriate behavior and rude advances. Despite such resistance and indignities, these young women remained committed to their duties. During a smallpox outbreak in Changsha, Hume dispatched nurse Li Guichen to the home of a prominent family. When the patriarch hesitated to accept her assistance, Hume reassured him: “They are educated women, trained to take care of the sick and to carry out the doctor's orders” (Hume 1946, 171–174). Even within the constraints of contemporary gender expectations, nurses played a critical role in Changsha's smallpox prevention and control efforts. Assumptions about the gendered division of nursing were reflected in the 1925 Nursing Policy Report, which stated that women were more suitable for bedside and community work, while men were preferred for administrative roles (Brundage et al. 1925). These assumptions reinforced rigid gender divisions and limited both women's leadership opportunities and men's participation in caregiving.

## 4 Challenges, Opportunities, and Limitations

Despite the progress made in training growing numbers of nurses, the Xiangya project unfolded amid shifting cultural and political dynamics that introduced challenges and tensions.

### 4.1 Cultural Hierarchies

The transnational spread of nursing education was not only a medical endeavor but also a cultural project shaped by implicit hierarchies of knowledge and civilization. One recurring theme was the “savior narrative,” which framed Americans as the sole bringers of nursing to China. Gage's remark that “In China, now, we are engaged in building up a nursing profession in a country which has not even a word for nurse in its language, let alone any conception of what the duties or advantages of a nurse or nursing service might be” overlooks indigenous caregiving traditions (Gage 1919). Additionally, traditional Chinese religious beliefs were often misunderstood or oversimplified, as Gage's characterization of Taoism makes clear: “Taoists consider that the patient has in some way incurred the wrath of one of the innumerable deities, or has been put under a spell by an enemy. Consequently, even nursing care might interfere with divine plans, and so except for priestly treatment, with or without a doctor's prescription, the patient, if too weak to walk, is generally left without anything more than mere waiting on” (Gage 1919). Gage's perspective failed to recognize the complexity and internal logic of local healing systems.

This perception of cultural superiority was not unique to American nurses' attitudes toward China but was instead characteristic of American nursing's global expansion in the 1920s (J. F. Irwin 2011). Across Europe, Asia, and the Caribbean, American nurses carried similar assumptions of cultural and racialized hierarchies, motivated by the desire “to tackle world health issues” and the conviction “that the spread of U.S. professional nursing ideas stood to modernize the world” (J. F. Irwin 2011, 78). In this sense, nursing education functioned as both a moral enterprise and a geopolitical instrument: the expansion of national and religious influence was the end, while the establishment of medical and nursing schools became the means to achieve it.

### 4.2 Political Instability

The Xiangya project was also shaped by the unstable political conditions of early 20th‐century China. During the 1910 revolutionary uprisings, the Xiangya mission was shielded from harm by the local community. In a moment recounted in Hume's memoirs, a Chinese family sheltered YCA personnel and their children during anti‐foreign riots. Hume later reflected on the improbability of such solidarity, writing, “It would have been hard to imagine American neighbors so hospitable to people of another race in the middle of the night, when fires were raging in nearby streets.” This act of quiet protection, offered at considerable personal risk, revealed the trust that the Xiangya project had cultivated among local residents (Hume 1946, 135). As Gage herself noted, the hospital's survival during the unrest may have been due to “a guiding authority in the rioting, or by the feeling of the people themselves toward foreign medical work” (Gage 1910).

In 1916, as civil unrest intensified, Gage reported that the hospital was inundated with victims of violence: “Of 90 patients now in the hospital, two typhoids and a few tubercular patients are the only exceptions to wounds by violence” (Gage 1916). Yet even in these volatile conditions, Gage maintained that nursing students gained critical hands‐on clinical experience that would have been unavailable in more controlled environments. During a period of strict curfews and heavily patrolled streets, Gage described being repeatedly stopped by armed soldiers while traveling in the city. Each time, the utterance of the phrase “Hsiangya” (Xiangya) prompted an immediate shift in tone: the soldiers would smile and wave her through with “pass friend,” reflecting a degree of recognition and respect for the institution's work (Gage 1916). These episodes illustrate that Xiangya's resilience in times of crisis rested not only on its foreign leadership but also on the strength of its community relationships and the cultural capital it had accrued through years of public health service.

### 4.3 Student Protests

Throughout its first decade, the school's leadership was also challenged by a series of student‐led strikes. Three days after the school opened, Gage received a petition signed by all 40 probationers demanding immediate instruction in materia medica, symptomatology, and the study of diseases. They protested having to wait 3 years before they could “begin to practice,” as the program required, and complained that the long delay kept them doing heavy ward work without access to clinical training. They also objected to night shifts, citing exhaustion and physical strain. By 1918, students organized a strike due to what they perceived as insufficient time off. On another occasion, classes had to be canceled because of a shortage of qualified instructors. One particularly dramatic incident involved a student ingesting bichloride of mercury in protest after her family pressured her to leave the program, revealing the personal and familial pressures that many trainees endured (Gage 1918).

The students' actions expose tensions between Western professional expectations and the practical realities of nursing education in China. They also make clear the agency and advocacy of the students asserting their own needs and priorities. For example, in summer 1924, Xiangya's men nurses and nursing students went on strike. The men explained they were protesting that the “work had never been what they had expected. Most of them had thought coming to the hospital meant studying medicine.” They were also frustrated that “the more unpleasant duties in a nurse's round are duties that should be performed by servants, not by professionally trained workers” (Xiangya Board 1925). For Xiangya's physicians, the strike was evidence that few of the men “understand and loyally accept the principles and ideals of the nursing project” (Xiangya Board 1925). The physicians resolved to “do away with men nurses” and focus only on the education of women nurses (Xiangya Board 1925). However, the men nursing students' strike was not an isolated event; it occurred in the context of student strikes at Yali Middle School and the College of Arts and Sciences, and was, as Holden explains, “part of a reaction sweeping China in 1924 and 1925 against foreigners and particularly against foreign educational institutions” (Holden 1964, 148). This burgeoning anti‐foreign sentiment led to the exodus of foreign missionaries and educators from China by 1927 (Lu et al. 2018).

## 5 Conclusion

By 1926, amid unprecedented anti‐foreign sentiment and the nationalization of Xiangya Medical and Nursing Schools, both Nina Gage and Edward Hume departed China. Three years later, Zigan Wang, assumed leadership of the nursing school, while also serving as the director of Xiangya Hospital. By the time of their departure, the Xiangya School of Nursing had trained 67 nurses—approximately 16% of China's total qualified nurses at the time. More importantly, the school had introduced a viable model for professionalized nursing education and provided a new vocational pathway for Chinese women who had only recently achieved suffrage. The institution remains in operation today, as a college affiliated with Central South University, and has become one of China's leading nursing school (“Xiangya School of Nursing, Central South University” n.d.).

The early history of the Xiangya School of Nursing contributes important insights to the scholarship on Chinese nursing history about the development of professional nursing in central China, highlighting the role of the YCA as a non‐denominational mission in building one of China's earliest undergraduate nursing programs. It also demonstrates the level of cultural negotiation and compromise Xiangya's nursing and medical leaders engaged in to adapt an Americanized model of nursing education to the local circumstances and needs of Changsha. By situating the school's early history in the context of similar transnational developments in China, it makes clear the distinct pathways to institutional growth among the earliest nursing schools, while also highlighting the interplay of transnational influence, cultural adaptation, and gendered reform in the development of nursing education in China in the early 20th century.

Beyond its historiographical significance, the history of Xiangya School of Nursing also offers enduring insights for contemporary transnational nursing education. First, it emphasized language acquisition—requiring American instructors to study Chinese—an approach that contrasts sharply with today's English‐dominant global education systems. Second, the concept of cultivating local leadership was essential. From its inception, the nursing education program emphasized the importance of developing and hiring Chinese nurse educators. Although this endeavor faced significant challenges, its prioritization in the planning process potentially helped the continuity of the program. Third, the history of Xiangya School of Nursing demonstrates the importance of cultural adaptation. Despite certain misunderstandings of local religious beliefs, educators like Gage and Hume made conscious efforts to localize care practices, such as substituting white quilts with red, and maintaining gender‐separated training. These adaptations were not superficial accommodations but stemmed from a genuine attempt to understand and translate the conceptual foundations of Chinese care traditions. These experiences speak to the program's deep social embeddedness.

Though rooted in a missionary and Western professional framework, the program gradually became a site of intercultural negotiation, co‐producing knowledge and hybridizing nursing identity. Its survival through political turmoil and its continued relevance as a premier institution underscore the transformative potential of long‐term, culturally responsive educational collaboration.

## 6 Limitation

A key limitation of this study lies in the nature of the available sources, which primarily reflect the perspectives of Edward H. Hume and Nina Gage. This inevitably shapes the analysis through the asymmetry of surviving records. Nevertheless, the study draws upon valuable archival materials, published primary documents, and relevant Chinese‐ and English‐language secondary sources to provide as balanced a perspective as possible.

## Funding

The authors have nothing to report.

## Ethics Statement

The authors have nothing to report.

## Conflicts of Interest

The authors declare no conflicts of interest.

## Data Availability

The data that support the findings of this study are openly available in Yale University Archives at https://archives.yale.edu/repositories/12/resources/2898.
